# A Bispecific Antibody to Link a TRAIL-Based Antitumor Approach to Immunotherapy

**DOI:** 10.3389/fimmu.2019.02514

**Published:** 2019-10-25

**Authors:** Alessandro Satta, Giulia Grazia, Francesco Caroli, Barbara Frigerio, Massimo Di Nicola, Francesco Raspagliesi, Delia Mezzanzanica, Nadia Zaffaroni, Alessandro Massimo Gianni, Andrea Anichini, Mariangela Figini

**Affiliations:** ^1^Biomarkers Unit, Department of Applied Research and Technical Development, Fondazione IRCCS Istituto Nazionale dei Tumori, Milan, Italy; ^2^Human Tumor Immunobiology Unit, Department of Research, Fondazione IRCCS Istituto Nazionale dei Tumori, Milan, Italy; ^3^Chemical Clinical Analysis Area, Laboratory Medicine Department, ASST Grande Ospedale Metropolitano Niguarda, Milan, Italy; ^4^Immunotherapy and Innovative Anticancer Therapeutics Unit, Department of Medical Oncology and Hematology, Fondazione IRCCS Istituto Nazionale dei Tumori, Milan, Italy; ^5^Oncological Gynecology Unit, Surgery Department, Fondazione IRCCS Istituto Nazionale dei Tumori, Milan, Italy; ^6^Molecular Therapies Unit, Department of Research, Fondazione IRCCS Istituto Nazionale dei Tumori, Milan, Italy; ^7^Molecular Pharmacology Unit, Department of Applied Research and Technical Development, Fondazione IRCCS Istituto Nazionale dei Tumori, Milan, Italy; ^8^Medical Oncology C Unit, Department of Medical Oncology and Hematology, Fondazione IRCCS Istituto Nazionale dei Tumori, Milan, Italy

**Keywords:** bispecific antibody, TRAIL-R2, immunotherapy, T-cell retargeting, malignant ascites

## Abstract

T-cell-based immunotherapy strategies have profoundly improved the clinical management of several solid tumors and hematological malignancies. A recently developed and promising immunotherapy approach is to redirect polyclonal MHC-unrestricted T lymphocytes toward cancer cells by bispecific antibodies (bsAbs) that engage the CD3 complex and a tumor-associated antigen (TAA). The TNF-related apoptosis-inducing ligand receptor 2 (TRAIL-R2) is an attractive immunotherapy target, frequently expressed by neoplastic cells, that we decided to exploit as a TAA. We found that a TRAIL-R2xCD3 bsAb efficiently activates T cells and specifically redirect their cytotoxicity against cancer cells of different origins *in vitro*, thereby demonstrating its potential as a pan-carcinoma reagent. Moreover, to mimic *in vivo* conditions, we assessed its ability to retarget T-cell activity in an *ex vivo* model of ovarian cancer patients' ascitic fluids containing both effector and target cells—albeit with a suboptimal effector-to-target ratio—with remarkable results.

## Introduction

T cell-based immunotherapy strategies have profoundly improved the clinical management of several solid tumors and hematological malignancies (1). The first, highly successful approach is the reactivation of tumor antigen-specific, MHC-restricted T cells through antibodies recognizing immune checkpoints (2). An alternative, but promising, possibility is to redirect polyclonal MHC-unrestricted T cells by chimeric antigen receptors (CAR-T cells) or by bispecific antibodies (bsAbs) that engage the CD3 complex (3).

The membrane receptor TNF-related apoptosis-inducing ligand receptor 2 (TRAIL-R2 or DR5), due to its up-regulation in many tumors compared to normal tissues (4, 5), could be considered a particular tumor-associated antigen (TAA) whose stimulation by its ligand (TRAIL) is able to transmit a death signal to the apoptotic machinery (6). The differential expression of the receptor along with its capacity to induce apoptosis specifically in tumor cells and preserve normal cells (7) opened a therapeutic window for the development of new cancer therapeutics (8–10). However, despite promising results in preclinical models, recombinant TRAIL or anti-TRAIL-R2 agonistic monoclonal antibodies (mAbs), failed to demonstrate anticancer activity in phase II randomized clinical trials (11, 12). To exploit the unique pattern of TRAIL-R2 expression, we developed a bsAb able to simultaneously bind TRAIL-R2 in an agonistic manner on cancer cells and the CD3-triggering molecule on T lymphocytes and demonstrated that this reagent could efficiently redirect their cytotoxicity against tumor cells *in vitro*.

Most importantly, we demonstrated these activities in an *ex vivo* model of ascitic fluids freshly isolated from ovarian cancer patients. Ascitic fluids present unique tumor microenvironment that is known exerts a prosurvival effect (13). Malignant ascites represent an unmet clinical need, associated with advanced disease and poor prognosis in different tumor types (14). Furthermore, ascites always contain a mixture of neoplastic and immune cells, including T cells (15), thus offering a unique opportunity to test the activity of our bsAb.

## Materials and Methods

### Cell Lines and Tissue/Cell Samples

Melanoma cell lines were established from surgical specimens of melanoma patients (stage IIIb to IV according to the American Joint Committee on Cancer) admitted to Fondazione IRCCS Istituto Nazionale dei Tumori, Milan, not previously treated. All lesions were histologically confirmed to be cutaneous malignant melanomas. The study was conducted in accordance with institutional guidelines and followed the principles of the Declaration of Helsinki. Melanoma cell lines were cultured in RPMI 1640 (BioWhittaker, Lonza—cat no BE12-702F) supplemented with 10% inactivated fetal bovine serum (FBS) of qualified USA origin (Gibco—cat no 26140-079), 2 mM L-glutamine (BioWhittaker, Lonza—cat no BE17-605E) and 20 mM HEPES buffer (BioWhittaker, Lonza—cat no 17737F) in a humidified chamber (95% air, 5% CO_2_) at 37°C. Main molecular and biological features of the cell lines used were published elsewhere (16). A2774 and NL-3507 epithelial ovarian carcinoma cells were gently provided by Dr Ferrini and Dr Van Der Burg, respectively. PC3, LNCaP, Du145 (prostate carcinoma), HepG2 (hepatocellular carcinoma), Caco-2 (colon carcinoma), A431 (epidermoid epithelial carcinoma), HeLa (epithelial adenocarcinoma of the cervix), SK-OV-3, A2780 (epithelial ovarian carcinoma), MDA-MB-231 and MDA-MB-468 (triple-negative breast cancer, TNBC), BT-474 (breast ductal carcinoma) and Jurkat (non-Hodgkin lymphoma) cell lines were purchased from the American Type Culture Collection (ATCC) and grown as indicated by the manufacturer. The hybridoma producing the anti-Myc-tag mAb 9E10 (CRL-1729) was purchased from ATCC and the hybridoma producing the anti-CD3 mAb TR66 was kindly provided by Prof. A. Lanzavecchia (17).

All cells were cultured for a maximum of 12 passages after thawing. To ensure the absence of mycoplasma contamination, all cell lines were routinely screened using a PCR Mycoplasma Test Kit I/C (PromoKine—cat no PK-CA91-1096) according to the manufacturer's instructions and genotyped at the functional genomic facility of our institute by means of the Promega StemElite ID System according to ATCC guidelines. Ovarian carcinoma tissues and ascites fluids were collected after all patients had signed an informed consent form, in accordance with the institutional ethics committee guidelines. Primary ovarian carcinoma cells were isolated from ascitic fluid samples of three chemotherapy-naïve patients at the time of primary surgery (13A, 15A, and 16A). Two short-term ovarian serous carcinoma cell lines (09ST and 10ST) were established from biopsies of two patients at the time of debulking surgery after three cycles of platinum-based chemotherapy. Cell lines from biopsies were established according to Guzzo et al. (18). For all primary cell lines and ascites-isolated cells, TRAIL-R2 expression was determined by flow cytometry, as described below.

Healthy donor buffy coats were provided by the Immuno-Hematology and Transfusion Medicine Unit of our Institute. Peripheral blood leukocytes (PBLs) were isolated from peripheral blood of healthy donors using a standard Ficoll density gradient centrifugation protocol (Ficoll-Paque^TM^ PLUS, GE Healthcare—cat no 17-1440-02), maintained in RPMI 1640 containing 10% pooled human serum (HS), and used for co-cultures within 24 h. For direct cytotoxicity assay, PBLs were activated using 150 IU Proleukin (Chiron Corporation, Novartis) for 4 days before use.

### Bispecific Antibodies

The human/humanized TRAIL-R2xCD3 bispecific single-chain diabody (scDb) (E7/UCHT1—patent number WO/2017/001681) was constructed as described (19).

The control scDb Mec14xCD3 contains the same antiCD3 moieties present in the TRAIL-R2xCD3 scDb but, with the other arm, could bind to the irrelevant herbicide mecoprep (Mec14) (20). The control scDb-gene was synthesized by Geneart (Thermo Fisher Scientific) and cloned in pIT2 vector.

Both scDbs were produced, purified and characterized as described (19).

### Tumor Cell Growth Inhibition and Cytotoxicity Assays

Tumor growth inhibition was evaluated by MTT (3-(4,5-dimethylthiazol-2-yl)-2,5-diphenyltetrazolium bromide) assay using PBLs as effectors and a panel of different cancer cell lines as targets. Twelve thousand tumor cells/well were plated in 96-well flat-bottom plates with the appropriate medium and incubated overnight. TRAIL-R2xCD3 scDb or Mec14xCD3 control scDb, at a concentration of 0.5 μg/mL, were then added and incubated for 1 h at 37°C before addition of non-activated PBLs at an effector-to-target (E:T) ratio of 5:1. After 48 or 96 h, the supernatant was removed and wells were gently washed three times with phosphate-buffered saline (PBS) to remove non-adherent PBLs. In each well 100 μL fresh medium containing 0.5 mg/mL MTT salt was added. After 3 h at 37°C, the supernatant was discarded and 150 μL of MTT solvent (isopropanol + 4 mM HCl + 0.1% NP40) was added. Absorbance at 590 nm (620 nm reference filter) was detected using a Bio-Rad 550 microplate reader. The percent tumor growth inhibition was calculated with respect to untreated cells. Where indicated, 1-h pre-incubation with 10 μg purified anti-TRAIL-R2 (BioLegend—cat no 307302) was used to block the binding of the scDb to tumor cells. The same MTT assay was used to test the sensitivity of cell lines to sTRAIL treatment: 2 × 10^4^ cells were incubated in the appropriate medium with 100 ng/mL sTRAIL at 37°C, 5% CO_2_. Tumor growth inhibition was assessed at 24 h.

Tumor cell cytotoxicity mediated by redirected PBLs was assayed by the calcein-AM (calcein-acetoxymethyl diacetyl ester) (BioVision Inc—cat no 1755-250) release assay (21), a method comparable to standard ^51^Cr release. One million target cells were resuspended in 1 mL complete medium containing the optimal concentration of calcein-AM, incubated for 30 min at 37°C, and washed three times with fresh medium. Ten thousand cells/well were seeded in 96-well round-bottom plates and incubated with the optimal concentration of TRAIL-R2xCD3 scDb, Mec14xCD3 control scDb or left untreated. Spontaneous release was determined by incubating calcein-AM-loaded cells with medium alone while maximum release was obtained incubating calcein-AM-loaded cells in medium containing 2% Triton X-100. After four to 16 h of incubation with pre-activated PBLs (E:T = 5:1), plates were centrifuged at 1,500 rpm for 10 min and the supernatant containing released fluorescent calcein was transferred to black-walled 96-well plates. The fluorescence intensity was measured using an ULTRA microplate reader (Tecan Group), with excitation/emission wavelengths of 485/535 nm. The cytotoxicity percentage was calculated as the percentage with respect to 100% lysis control.

### Flow Cytometry Experiments

Detection of the scDb recognition capacity was performed by FACS analysis as described by Satta et al. (19). T-cell activation following scDb incubation was assessed by expression of activation antigen, T-cell degranulation, and T-cell proliferation. Expression of surface activation antigens and intracellular molecules was evaluated by multicolor flow cytometry on a Gallios flow cytometer (Beckman Coulter) as previously described (22). When needed, cell permeabilization was obtained with a Cytofix/Cytoperm Fixation/Permeabilization kit (BD Pharmingen—cat no 554714) according to the manufacturer's instructions. Briefly, 2 × 10^6^ lymphocytes were resuspended in 100 μL of staining buffer (PBS plus 2% FBS) and stained for 30 min at +4°C with antibodies for cell surface markers; cells were then washed with cold PBS and fixed for 20 min on ice with the fixation solution; after fixation, cells were washed twice with Perm-wash and stained with the appropriate intracellular antibodies. Where indicated, 1-h pre-incubation with 10 μg purified anti-TRAIL-R2 (BioLegend—cat no 307302) or pre-coated ultra-LEAF purified anti-CD3 (10 μg; BioLegend—UCHT clone; cat no 300413) was used as controls.

In functional assays evaluating lymphocyte degranulation, the experiments were performed in RPMI 1640, 10% HS, in the presence of PE-CD107a (10 μg/mL); GolgiStop (BD Biosciences—cat no 554724) was added 1 h after the start of the co-culture. After 5 h cells were collected and stained as described above.

For proliferation experiments PBLs were initially stained with carboxyfluorescein succinimidyl ester (CFSE) (Molecular Probes-Thermo Fisher Scientific—cat no C34554) as described (23), then cultured with melanoma or breast cancer cells in the presence or absence of the indicated doses of scDb and stained as described above. Lymphocyte stimulation with phytohaemagglutinin (PHA, 5 μg/mL) was used as positive control. Quantification of apoptotic cells following incubation with scDb and effectors was carried out by flow cytometry after staining with APC-conjugated annexin V (BD Pharmingen—cat no 550474) and propidium iodide (BD Biosciences—cat no 556463) as previously described (23). For a complete list of the antibodies used, see Table 1. Analyses were performed with the FlowJo software v. 10.1 and data were gated on live cells, after the exclusion of doublets.

**Table 1:** Antibodies used for flow cytometry.

**Antigen**	**Fluorochrome**	**Clone**	**Company**	**Code**
CD107a	PE	H4A3	BD Biosciences	555801
CD137	PE	4B4-1	Miltenyi Biotec	130-093-475
CD137	PE-Dazzle594	4B4-1	BioLegend	309826
CD14	ECD	RMO52	Beckman Coulter	IM2707U
CD19	APC	HIB19	BD Biosciences	555415
CD25	FITC	2A3	BD Biosciences	345796
CD25	APC	2A3	BD Biosciences	340907
CD279 (PD-1)	BV421	EH12.2H7	BioLegend	329920
CD3	APC-H7	SK7	BD Biosciences	560176
CD4	PerCP-Cy5.5	RPA-T4	BD Biosciences	560650
CD4	PE-Cy7	RPA-T4	BD Biosciences	560649
CD45	APC-Alexa Fluor 700	J33	Beckman Coulter	A79390
CD45	BV421		BD Biosciences	563879
CD69	PE-Cy7	FN50	BD Biosciences	557745
CD8	BV510	SK1	BD Biosciences	563919
CD8	PerCP-Cy5.5	RPA-T8	BD Biosciences	560662
HLA-DR	FITC	G46-6	BD Biosciences	
IFN-γ	FITC	B27	BD Biosciences	554700
Granzyme B	Alexa Fluor 647	GB11	BD Biosciences	560212
Perforin	PE	dG9	eBioscience	12-9994
Zombie Aqua			BioLegend	423102

### Immunofluorescence Experiments

To test the scDb-mediated redirection of T cells, they were stained using a PKH26 Red Fluorescent Cell Linker Kit (Sigma-Aldrich—cat no PKH26GL-1KT), according to the manufacturer's instructions and co-incubated with Me15 cell line (stained with calcein-AM as described above). After 4 h of treatment the T-cell/Me15 co-localization was assessed. Mec14xCD3 scDb was used as control.

ScDb activity on ovarian cancer cells was assayed treating tumor cells for 16 h with scDb directly in ascitic fluid. After 16 h treatment, cells were stained with anti-EpCAM mAb (Table 1) and propidium iodide (BD Biosciences—cat no 556463) as described.

Samples were analyzed using an inverted microscope with a 20X Plan Fluor objective (Nikon) and acquired with ACT-1 software (Nikon). Quantification of co-localization was performed using ImageJ and JACoP v2.0 as described (24).

### Statistical Analysis

The GraphPad Prism software was used to generate graphs and perform statistical analysis. Student's *T*-test and One-way ANOVA followed by multiple comparison testing was carried out to determine the significance of differences between treatments.

## Results

### TRAIL-R2/CD3 scDb Antitumor Activity

The scDb used in this paper was previously selected from a library in order to obtain a small molecule able to simultaneously bind TRAIL-R2 on tumor cells and the CD3 molecule on T lymphocytes (19). Three melanoma cell lines expressing different levels of TRAIL-R2 (Me15, Me41, and Me64) and two breast cancer cell lines (MDA-MB-468 and BT-474) totally negative for the receptor (Table 2) were used to confirm the correct binding of our antibody. As expected given its monovalent binding, our scDb was able to similarly bind the melanoma cell lines to the commercially available positive control antibody (bivalent entire IgG) while remaining negative on MDA-MB-468 and BT-474 cells (Supplementary Figure 1). ScDb binding to the CD3 specificity was assessed on Jurkat, an immortalized human T lymphocytes cell line (Supplementary Figure 1).

**Table 2:** Tumor cell line characterization.

**Tumor type**	**Cell line**	**TRAIL-R2-relative MFI^a^**	**sTRAIL sensitivity^b^**
Melanoma	Me15	103.8	Sensitive
	Me41	103.8	Semi-sensitive
	Me64	3.8	Resistant
	Me71	17.5	Resistant
	Me79	42.5	Resistant
Prostate carcinoma	PC3	111.5	Sensitive
	LNCaP	603.3	Resistant
	DU145	694.0	Sensitive
Ovarian carcinoma	SK-OV-3	8.4	Resistant
	NL-3507	7.1	Sensitive
	A2780	11.9	Resistant
	A2774	16.4	Semi-sensitive
Cervix carcinoma	HeLa	3.4	Resistant
Epidermoid carcinoma	A431	6.7	Semi-sensitive
Hepatocellular carcinoma	HepG2	168.0	Sensitive
Colon carcinoma	Caco-2	308.1	Resistant
Breast carcinoma	MDA-MB-231	10.3	Sensitive
	MT-3	12.2	NA
	MDA-MB-468	−0.1	Sensitive
	BT-474	0.1	Resistant

a *MFI, mean fluorescence intensity evaluated by FACS analysis*.

b *Evaluated by tumor growth inhibition assay using 100 ng/mL sTRAIL. The growth inhibition percentage allows to distinguish resistant (<30%), semi-sensitive (30–50%) and sensitive (>50%) cell lines. NA, not available*.

**Figure 1:**
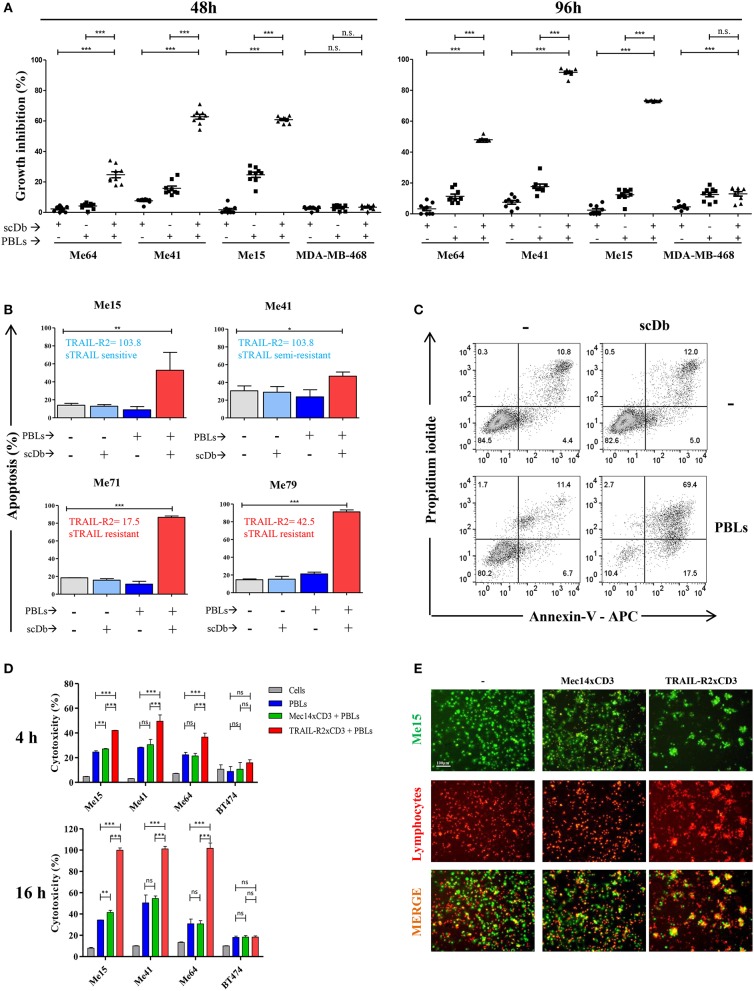
ScDb-redirected T-cell killing of melanoma cell lines. **(A)** Growth inhibition assays performed using nine different batches of PBLs (each derived from a different healthy donor) in the presence or absence of scDb. TRAIL-R2-expressing Me64, Me41, and Me15 and TRAIL-R2-negative MDA-MB-468 were used as target cells treated with 0.5 μg/mL of scDb and non-activated PBLs at an E:T ratio of 5:1 for 48 h (left panel) or 96 h (right panel). Each point represents a single experiment. Bars represent the mean of all nine experiments; error bars, SD. **(B)** Percentage of apoptosis (early + late apoptosis, annexin V^+^) of two sTRAIL-sensitive melanoma cell lines (top, Me15 and Me41) and two sTRAIL-resistant melanoma cell lines (bottom, Me71 and Me79) co-cultured for 72 h with 0.1 μg/mL scDb, non-activated PBLs, or both at E:T = 5:1. (*n* = 3; error bars, SD). **(C)** Representative plot of data shown in **(B)**. **(D)** Tumor cells were treated for 4 and 16 h with pre-activated PBLs in the presence or absence of TRAIL-R2xCD3 scDb. Irrelevant scDb Mec14xCD3 was used as control. The graphs show the percentage of direct cell lysis as the mean ± SD of three experiments. **(E)** Immunofluorescence was performed using Me15 cells loaded with calcein-AM (green) and grown for 4 h with pre-activated PBLs stained using a PKH26 Red Fluorescent Cell Linker Kit in the absence (left panels) or presence (right panels) of 0.5 μg/mL TRAIL-R2xCD3 scDb. 0.5 μg/mL irrelevant scDb Mec14xUCHT1 was used as control (central panels). The pictures were taken at the end of the incubation time. Statistical analysis in **(A,B,D)** by one-way ANOVA followed by Tukey's post-test. ^*^p < 0.05, ^**^p < 0.01, ^***^p < 0.001; ns: not significant.

The same melanoma and breast cancer cell lines were used to test the ability of the scDb to specifically retarget PBLs. Not previously activated PBLs from nine different healthy donors were co-cultured with the cancer cell lines in the presence or absence of our scDb. Using 0.5 μg/mL scDb and an E:T ratio of 5:1, significant tumor growth inhibition was obtained in TRAIL-R2-expressing tumor cells with all the PBL preparations tested and with acceptable allogeneic PBL activity, both at 48 and 96 h of incubation (Figure 1A).

Melanoma cell lines used were selected on the basis of their different sensitivity to sTRAIL (Table 2). Annexin-V/propidium iodide staining showed that non pre-activated PBL incubation for 72 h with 0.1 μg/mL scDb at an E:T ratio of 5:1 can bypass the sTRAIL resistance of cancer cells, achieving a good apoptosis percentage in all melanoma cell lines tested, independent of their susceptibility profile to sTRAIL (Figures 1B,C). Similar results were obtained in cytotoxicity experiments where pre-activated PBLs efficiently lysed target cells when the scDb was present in the co-culture (Figure 1D).

Importantly, in order to confirm the ability of our scDb to promote the formation of effector-target conjugates, we labeled pre-activated T cells using a PKH26 Red Fluorescent Cell Linker Kit and stained Me15 target cells with calcein-AM. As shown in Figure 1E, co-localization of effector and target cells was evident after 4 h of co-incubation only in the presence of the scDb; of note, the disappearance of target cells confirmed the killing activity of redirected PBLs.

**Figure 2:**
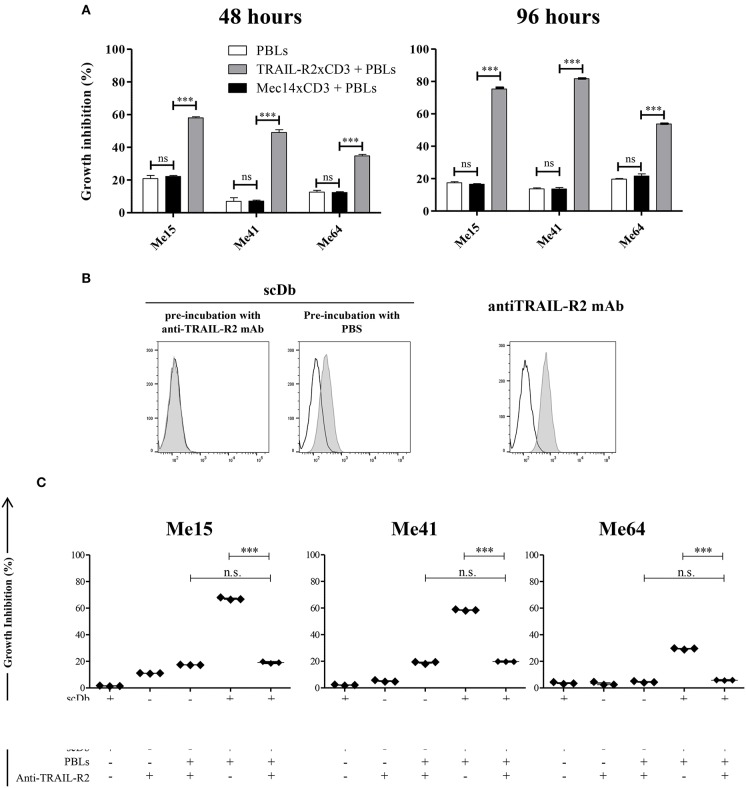
Specificity of scDb-mediated cytotoxicity. **(A)** Growth inhibition assays were performed treating Me15, Me41, and Me64 cells with 0.5 μg/mL irrelevant scDb Mec14xCD3 or scDb TRAIL-R2xCD3 and non activated PBLs at an E:T ratio of 5:1 for 48 and 96 h. Mean values and SD of triplicate determinations are shown. **(B)** Competition between a commercial anti-TRAIL-R2 mAb and the scDb was evaluated by FACS. Before the addition of the scDb, TRAIL-R2^+^ Me41 cells were incubated for 1 h with the anti-human TRAIL-R2 mAb (left panel) or with PBS (middle panel). Binding of the scDb is revealed by an anti-6xHis tag rabbit mAb followed by an Alexa Fluor 488-labeled anti-rabbit Ab. The binding of the competing anti-TRAIL-R2 mAb was revealed by a secondary Alexa Fluor 488-labeled antimouse Ab (right panel). Empty peaks: negative control; gray peaks: scDb or mAb. **(C)** Growth inhibition assays were performed treating Me15, Me41, and Me64 cells with 0.5 μg/mL scDb and non-activated PBLs at an E:T ratio of 5:1 for 48 h. Pre-incubation with 10 μg/mL anti-TRAIL-R2 mAb was used as scDb-competing mAb to test scDb specificity. The graphs show the mean ± SD of three experiments. Each point represents a single experiment. Bars represent the mean ± SD. Statistical analysis in **(A,C)** by one-way ANOVA followed by Tukey's post-test. ^***^p < 0.001; n.s.: not significant.

No effect was observed against TRAIL-R2-negative MDA-MB-468 and BT-474 cells (Figures 1A,D), confirming the specificity of the scDb. A negative control scDb, having the same anti-CD3 specificity but recognizing an irrelevant antigen was produced, purified and tested for its binding ability to TRAIL-R2^+^ or CD3^+^ cells (Supplementary Figures 2A,B). This negative control scDb was unable to redirect PBL activity against TRAIL-R2^+^ melanoma cell lines, thus confirming the specificity of the TRAIL-R2xCD3 scDb effect (Figures 1D,E, 2A). Importantly, a pre-incubation with an antiTRAIL-R2 mAb, which competed for the same binding site of scDb on TRAIL-R2 (Figure 2B), was sufficient to revert the growth inhibitory effect on melanoma cell lines obtained by PBLs in the presence of the bispecific antibody, further confirming the specificity of the effect seen (Figure 2C).

**Figure 3:**
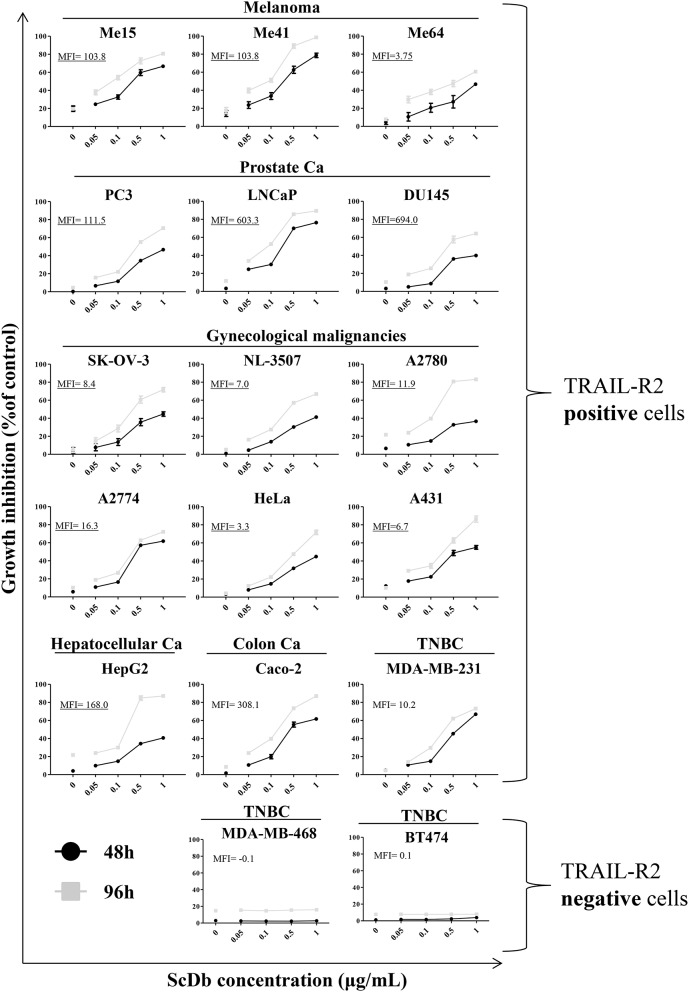
Tumor growth inhibition assay on a panel of TRAIL-R2-positive or negative cells. ScDb-dependent PBL-mediated growth inhibition was performed on several cancer cell lines using scDb concentrations of 0, 0.05, 0.1, 0.5, and 1 μg/mL and an E:T ratio of 5:1 for 48 h (black line) or 96 h (gray line). Non-activated PBLs were used as effectors. The graph represents the mean ± SD of three experiments. In each graph the expression level of TRAIL-R2 as mean fluorescence intensity (MFI) is reported.

We then decided to test the activity of the scDb on a panel of TRAIL-R2^+^ tumor cell lines of different origins and characterized by different TRAIL-R2 expression levels as well as different sensitivity to sTRAIL (Table 2). ScDb-dose-dependent tumor growth inhibition was observed with an E:T ratio of 5:1 at both 48 and 96 h of incubation while no effect were seen on TRAIL-R2 negative MDA-MB-468 and BT-474 cells (Figure 3). PBLs alone caused a weak cytotoxic effect that exceeded 10% of growth inhibition only in the A2780 and HepG2 cell lines after 96 h of treatment (Figure 3).

### ScDb-dependent T-Cell Activation

**Figure 4:**
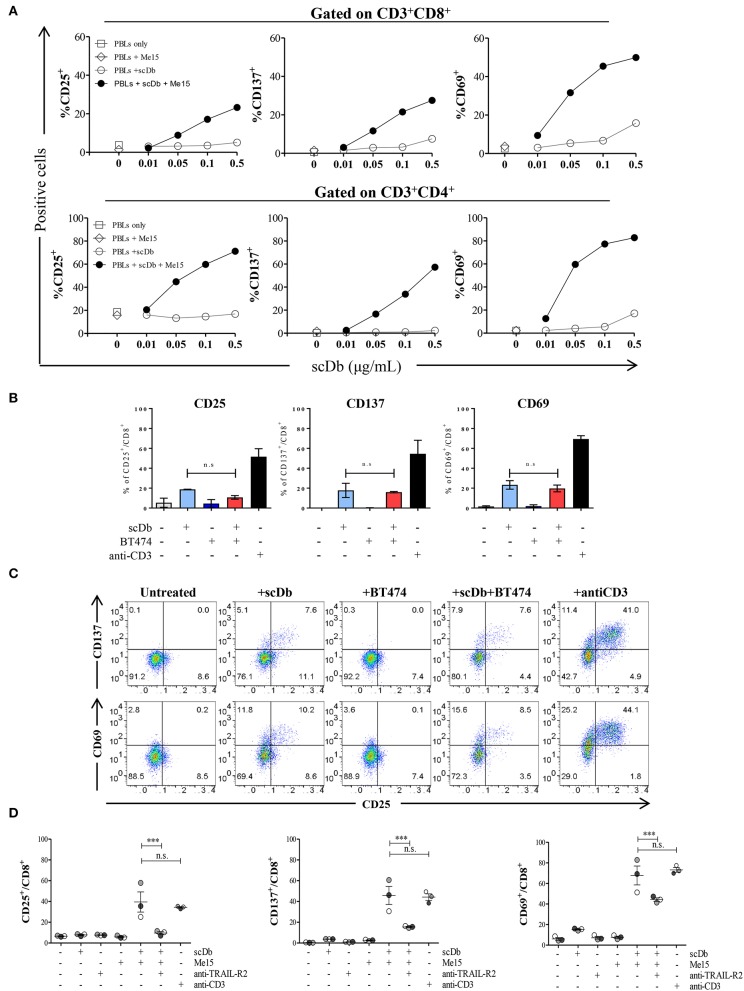
Effects of scDb on T-cell activation. **(A)** Frequency of CD25^+^, CD137^+^, and CD69^+^ T lymphocytes in CD8^+^ (top) and CD4^+^ (bottom) subsets after treatment with increasing doses of scDb in the presence of TRAIL-R2^+^ Me15 cells at E:T=5:1. **(B)** Effects of scDb on T-cell activation when TRAIL-R2 negative BT-474 cells were treated. Frequency of CD25^+^, CD137^+^, and CD69^+^ T lymphocytes in CD8^+^ subsets after treatment with 0.5 μg/mL scDb in the presence of TRAIL-R2^−^ BT-474 cells at E:T=5:1. Pre-incubation with 10 μg/mL anti-CD3 was used as positive control. The graphs show the mean ± SD of three experiments. **(C)** Representative plot of **(B)**. **(D)** Frequency of CD25^+^, CD137^+^, and CD69^+^ cells in CD8^+^ T lymphocytes (after gating on CD45^+^CD3^+^ cells) after 16 h of treatment with 0.5 μg/mL scDb in the presence or absence of the melanoma target cell line (Me15). Pre-incubation with 10 μg/mL anti-TRAIL-R2 mAb and anti-CD3 was used as scDb-competing Ab and positive control, respectively. The graphs show the mean ± SD of three experiments. Statistical analysis in **(B,D)** by one-way ANOVA followed by Tukey's post-test. ^***^p < 0.001; ns: not significant.

Upon simultaneous binding to TRAIL-R2^+^ target cells and CD3^+^ T lymphocytes, the scDb should induce the formation of an immune synapse with consequent activation of T cells. To confirm this hypothesis, we measured the expression of the classical T-cell activation markers CD25, CD137 and CD69 by FACS after 16 h of PBLs co-culture with TRAIL-R2^+^ Me15 cells in the presence or absence of the scDb at increasing concentrations. The scDb induced activation of both CD8^+^ (Figure 4A, top) and CD4^+^ (Figure 4A, bottom) T cells in a dose-dependent manner, as evidenced by up-regulation of all the markers tested. Further experiments with additional melanoma cell lines (Me41, Me64) suggested no evident correlation between TRAIL-R2 expression levels and extent of T cell activation (Supplementary Figure 3), while no activation was seen in the presence of TRAIL R2 negative BT-474 cell line (Figures 4B,C). Importantly, competition experiments with a commercial antibody directed to TRAIL-R2 significantly inhibited the scDb-dependent up-regulation of CD25, CD137, and CD69 in T lymphocytes co-cultured with melanoma target cells (Figure 4D), indicating that T-cell activation requires the scDb to bind to both specificities. In these experiments stimulation with bivalent anti-CD3 provided effective T-cell stimulation.

**Figure 5:**
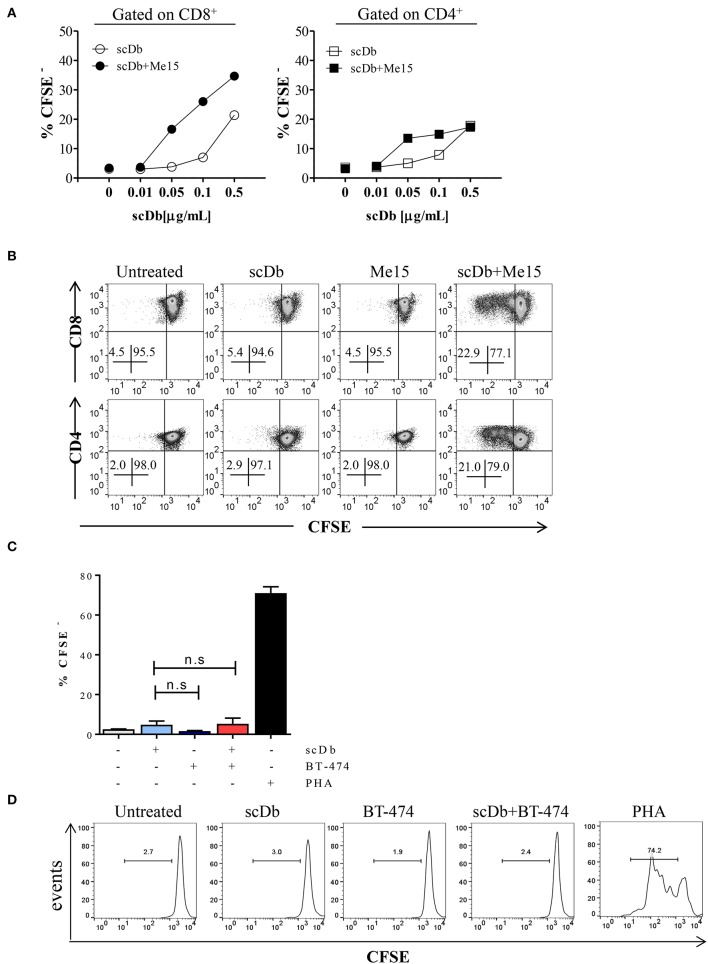
ScDb-mediated CD4^+^ and CD8^+^ T-cell proliferation. **(A)** Proliferation (CFSE dilution assay) of CD8^+^ and CD4^+^ T cells after overnight treatment with increasing doses of scDb in the presence or absence of TRAIL-R2^+^ melanoma target cells. **(B)** Representative plot of data shown in **(A)**. **(C)** Proliferation assay of CD8^+^ T cells at day +4 after treatment with 0.1 μg/mL scDb in the presence or absence of TRAIL-R2 negative BT-474 cells. Stimulation with PHA (5 μg/mL) was used as positive control. Graph represents mean ± SD of three different experiments. **(D)** Representative plot of **(C)**. Statistical analysis in **(C)** by one-way ANOVA followed by Tukey's post-test. ns: not significant.

Moreover, we demonstrated that co-culture of non-activated PBLs with TRAIL-R2^+^ melanoma cells in the presence of the scDb also led to activation of CD4^+^ and CD8^+^ T-cell proliferation, as shown by CFSE assay (Figures 5A,B), but not of CD3^−^/CD19^+^ cells (Supplementary Figure 4). Again, no proliferation of CD8+ T lymphocytes was observed using the receptor negative BT-474 for co-culture experiments (Figures 5C,D).

**Figure 6:**
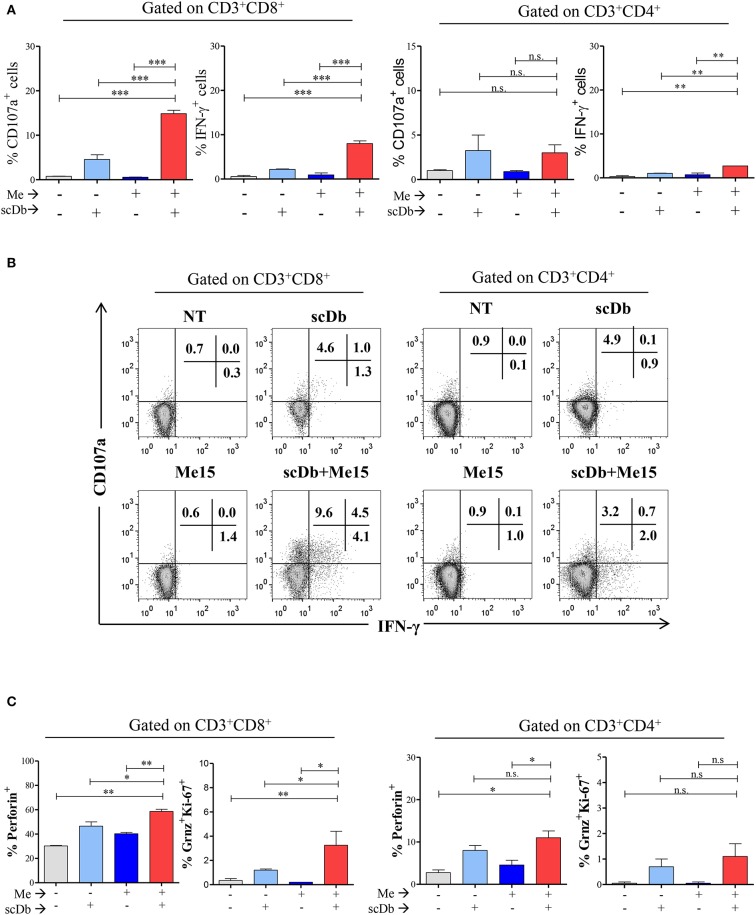
ScDb triggering of PBL lytic machinery. **(A)** Increased frequency among CD3^+^/CD8^+^ and CD3^+^/CD4^+^ T lymphocytes of CD107a^+^ or IFN-γ^+^ cells after treatment with 0.1 μg/mL scDb in the presence or absence of a melanoma target cell line (Me15) at E:T 5:1. The graphs represent the mean ± SD of three experiments. **(B)** Surface expression of CD107a and intracellular staining for IFN-γ by flow cytometry in CD3^+^/CD8^+^ (left) and CD3^+^/CD4^+^ (right) T-cell subsets after treatment with 0.1 μg/mL scDb in the presence or absence of the Me15 melanoma target cell line. **(C)** Frequency among CD3^+^/CD8^+^ (left graphs) and CD3^+^/CD4^+^ (right graphs) T lymphocytes of perforin, granzyme B and Ki-67 expression, after treatment with 0.1 μg/mL scDb in the presence or absence of a melanoma target cell line (Me15) at E:T 5:1. The graphs represent the mean ± SD of three experiments. Statistical analysis in **(A,C)** by one-way ANOVA followed by Tukey's post-test. ^*^p < 0.05, ^**^p < 0.01, ^***^p < 0.001; ns: not significant.

A further confirmation of the activation status of T lymphocytes in the presence of the scDb was obtained by evaluating the expression of the degranulation marker CD107a and the production of interferon-gamma (IFN-γ); significant upregulation of both markers in CD8^+^ T cells was seen in co-culture experiments only when the scDb was present (Figures 6A,B).

Importantly, our scDb was able to render CD8^+^ T lymphocytes cytotoxic, as evidenced by the increase in the percentage of perforin-positive cells and T lymphocytes double-positive for granzyme B and Ki-67 (Figure 6C).

### Redirecting Autologous Lymphocytes Against Ovarian Cancer Cells

**Figure 7:**
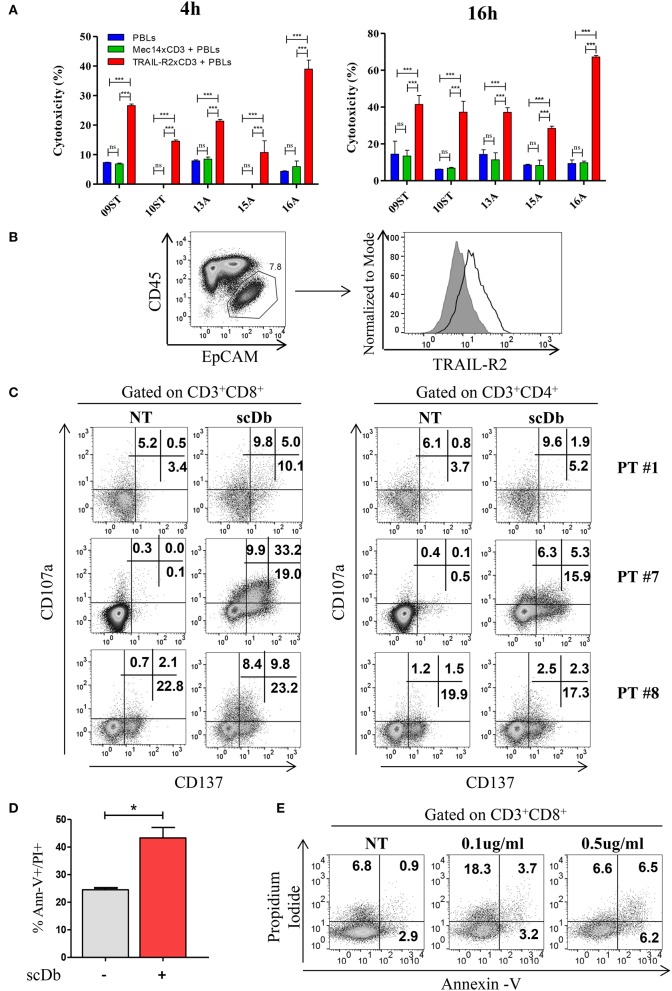
Treatment of *ex vivo* cells derived from ovarian cancer patients. **(A)** Primary ovarian carcinoma cytotoxicity assay after 4 and 16 h of treatment with TRAIL-R2xCD3 or Mec14xCD3 scDbs and pre-activated healthy donors' PBLs. 09ST and 10ST: short-term ovarian cancer cell lines established from biopsies; 13A, 15A, and 16A: cells isolated from ascitic fluid of ovarian cancer patients. The graphs show the percentage of direct cell lysis as the mean ± SD of three wells for treatment. Statistical analysis by one-way ANOVA followed by Tukey's post-test. ^***^p < 0.001; ns: not significant. **(B)** Representative staining for CD45 and EpCAM (left) and TRAIL-R2 expression in EpCAM^+^ tumor cells (right) in an ascitic fluid from an ovarian cancer patient. **(C)** Flow cytometry expression of the surface activation markers CD107a and CD137 on CD8^+^ (left) and CD4^+^ (right) T lymphocytes of ascites samples from three ovarian cancer patients, treated or not with scDb (0.5 μg/mL, 18 h). **(D)** Annexin-V/propidium iodide apoptosis assay of tumor cells (EpCAM^+^/CD45^−^) treated or not with 0.5 μg/mL scDb for 48 h. Statistical analysis by Student *T*-test. ^*^p < 0.05. **(E)** Annexin-V/propidium iodide apoptosis assay on CD8^+^ T cells treated as in **(D)**.

We then decided to test the activity of our antibody using five *ex vivo* short-term cultures of neoplastic cells derived from tumor biopsies or freshly collected from ascitic fluids of ovarian carcinoma patients. These short-term cultures were initially characterized for EpCAM and TRAIL-R2 expression by FACS, and showed different expression levels of the death receptor (Table 3). The treatment for 4 and 16 h with 0.5 μg/mL scDb was able to mediate the redirection of pre-activated healthy donors' PBLs to lyse tumor cells; Mec14xCD3 scDb, used as control, provoked levels of cytotoxicity comparable to PBLs alone confirming the specificity of the scDb-activity (Figure 7A).

**Table 3:** Clinicopathological characterization of samples from ovarian cancer patients.

**Patient ID**	**Tumor histology^a^**	**Surgery^b^**	**EpCAM expression**		**TRAIL-R2 expression**	
			**ST^c^**	**A^d^**	**ST^c^**	**A^d^**
09	Serous	Interval surgery	+++	/	++	/
10	Serous	Interval surgery	+++	/	+	/
13	Papillary serous	Primary surgery	/	+++	/	+++
15	Serous	Primary surgery	/	+++	/	++
16	Serous	Primary surgery	/	+++	/	++

a *All ovarian cancers were grade 3*.

b *Samples obtained after interval surgery had been exposed to chemotherapy; those obtained at primary surgery were chemo-naïve*.

c *ST, ex vivo cells derived from solid tumors*.

d *A, ex vivo cells derived from ascites*.

*/, Not available*.

*+, low expression; ++, average expression; +++, high expression*.

Similar results were obtained in ScDb-mediated tumor growth inhibition experiments using non-activated patients' autologous PBLs (Supplementary Figure 5).

To obtain proof of principle of a clinically relevant application, we tested the scDb activity on ovarian cancer cells in their own ascitic fluid, which usually contains also effector cells and represents the real tumor microenvironment. We characterized fresh ovarian cancer ascitic fluid samples for the presence of cancer cells and T cells. FACS analysis showed that CD45^−^/EpCAM^+^ neoplastic cells accounted for percentage between 0.5 and 15% of the total and stained positive for TRAIL-R2 expression (Figure 7B and Table 4). Treatment with 0.5 μg/mL scDb for 16 h resulted in the up-modulation of CD107a and CD137, mostly in the CD8^+^ cells present in ascitic fluids of three different patients (Figure 7C), and in increased cytolytic activity on CD45^−^/EpCAM^+^ neoplastic cells, as demonstrated by double-positive staining with annexin V and propidium iodide (Figure 7D). Of note, the specificity of the effect was confirmed also in this model because the addition of an excess of anti-TRAIL-R2 mAb prevented the up-modulation of the degranulation marker CD107a on T CD8^+^ lymphocytes (Supplementary Figures 6A,B). Control assays showed that ascitic fluid T cell activation (detected as increase in CD107a^+^/CD137^+^ cells in the lymphocyte-tumor-scDb co-cultures) could be induced in CD8^+^ T cells only by the TRAIL-R2xCD3 scDb, but not by the Mec14xUCHT1 scDb (Supplementary Figure 7).

**Table 4:** Characterization for tumor cells presence and for TRAIL-R2 expression of three ovarian malignant ascites.

**Malignant ascites ID**	**% tumor cells (EpCAM^**+**^CD45^**−**^)**	**% TRAIL-R2^**+**^ tumor cells**
PT #1	7.8	19.2
PT #7	0.5	35.0
PT #8	15.0	70.3

A significant increment in tumor cell death was confirmed also in immunofluorescence experiments where EpCAM positive cells become propidium positive when treated with scDb (Supplementary Figures 8A,B). Importantly, only very weak T-cell self-killing was observed after treatment (Figure 7E).

## Discussion

Immunotherapy is one of the most exciting fields of cancer research today. In the last decades many efforts have been made to take advantage of the potency of T cells in cancer therapy, even by redirecting them against TAAs independent of the T-cell-receptor-defined specificity (3). The best TAA would be the one giving the best therapeutic window between the tumor target and normal tissues, and with this in mind we decided to use TRAIL-R2 as a TAA. The selectivity of TRAIL-R2 is mainly due to the fact that its triggering by TRAIL or TRAIL-agonistic mAbs has an apoptotic effect on cancer cells without the lethal adverse effect on normal cells that has been observed following activation of other death receptors like CD95 (7, 25). Here we describe and characterize a bsAb, in scDb format, that redirects T-cell cytotoxic activity through engagement of CD3 against tumor cells; we were the first to use TRAIL-R2 as a TAA. Using phage display technology, we obtained a stable and specific reagent able to redirect T cells against tumor cells of different origins and therefore having the characteristics of a potentially pan-cancer reagent. The scDb was able to efficiently activate T cells and to overcome tumor cell resistance to sTRAIL-mediated cytotoxicity. More importantly, we provided proof of principle of its activity in an *ex vivo* model mimicking a real clinical setting.

Remarkable progress in bsAb technology has been made during the last decade and more than 50 different methods to build bsAbs have been described in the literature (26). In December 2014, the tascFv bispecific T-cell engager (BiTE) blinatumomab, directed against CD19 and CD3 to retarget T lymphocytes against CD19^+^ cells, was approved by the US Food and Drug Administration under the accelerated approval program for the treatment of acute lymphoblastic leukemia (27). The success of the first-generation BiTE antibodies has led to an explosive clinical development of T-cell-redirecting bsAbs. One novelty of the product described here is that the redirection of T cells is against a non-conventional TAA, TRAIL-R2. Although the agonistic activity of scDb is low (19), this activity can be combined with its powerful ability to retarget T cells against TRAIL-R2^+^ tumor cells, resulting in efficient lysis of all treated tumors. Furthermore, the capacity to induce T-cell-mediated cytotoxicity also against sTRAIL-resistant target cells allows the scDb to overcome the ability of cancer cells to develop resistance to the extrinsic apoptosis pathway. Interestingly the bsAb failed to activate T cells and to promote tumor cell killing only when neoplastic cells where TRAIL-R2-negative, while the bsAb was effective even when the target cells expressed low levels of TRAIL-R2. Furthermore, the cytotoxic effect provoked by scDb-retargeted T-cells does not correlate with the expression of the target on tumor cells surface. This suggests that even a low level of expression of TRAIL-R2 (for example in Me64) may be sufficient to induce the immunological synapse (bridging CD3 and TRAIL-R2 through the bsAb) needed for T cell activation and subsequent anti-tumor functions in lymphocyte-tumor co-cultures where the bsAb is added.

The scDb could activate T cells without the addition of exogenous stimulation and probably following the formation of the immunocytolytic synapse. Interestingly, we found that T-cell activation, as documented by up-regulation of CD25, CD137, and CD69, by the TRAIL-R2/CD3 scDb was strictly dependent on the presence of TRAIL-R2^+^ tumor cells in the experimental assay. Furthermore, preventing scDb interaction with TRAIL-R2^+^ tumor cells through a competing anti-TRAIL-R2 antibody, we found that T-cell activation was abolished. Collectively, these data provide evidence for the remarkable TRAIL-R2 specificity of the scDb and suggest that off-target T-cell activation may be avoided, which is a highly relevant issue for *in vivo* bsAb translation.

In addition to providing effective signals for TRAIL-R2-dependent early T-cell activation, the scDb was able to promote T-cell proliferation and functional differentiation, in agreement with the functional features of different recently described CD3 bsAbs (28, 29). We found that both CD4^+^ and CD8^+^ T cells, but not control B cells, proliferated in response to the scDb in the presence of TRAIL-R2^+^ melanoma cells. Furthermore, under these experimental conditions, the scDb also promoted T-cell differentiation to the cytolytic stage (up-regulation of perforin and granzyme B and surface expression of the degranulation marker CD107a) associated with IFN-γ production. These effects on the functional differentiation of T cells were mostly observed in the CD8^+^ subset, suggesting that the scDb is highly effective in the generation of cytolytic CD8^+^ T cells, which are key antitumor effectors in immunotherapy.

We obtained *in vitro* proof of principle that the scDb we developed could be a potentially useful therapeutic option for a variety of tumors. However, in the instance of solid tumors, efficacy of the bsAb therapeutic approach may depend on a variety of factors, including the need for a pre-existing T cell infiltrate in order to achieve the anti-tumor activity expected (27). Therefore, a more feasible clinical development of the bsAb described in this study could be in the setting of loco-regional treatments (peritoneal cavity administration) for ovarian cancer patients. Ascites always contain a mixture of neoplastic and immune cells, including T cells, and in patients with malignant ascites there is already pre-clinical and clinical evidence for the activity of the EpCAM/CD3 bispecific antibody Removab (30, 31). The withdrawal of this bsAb from the market maintains open the necessity of a treatment for malignant ascites. We were able to demonstrate the ability of the scDb to efficiently redirect T cells against TRAIL-R2-expressing ovarian cancer cells, simply adding the soluble scDb to malignant ascites and thus mimicking a real human *in vivo* situation. We are aware that lack of an *in-vivo* solid tumor model limits the potential translational relevance of the scDb described in this study. Nevertheless, we believe that the results of our work can support a pre-clinical rationale for a new therapeutic option in patients with malignant ascites, where new treatments are urgently needed.

## Data Availability Statement

The raw data supporting the conclusions of this manuscript will be made available by the authors, without undue reservation, to any qualified researcher.

## Ethics Statement

The study was conducted in accordance with institutional guidelines and followed the principles of the Declaration of Helsinki. The study protocol was approved by the local Ethics Committee of the Fondazione IRCCS Istituto Nazionale dei Tumori, Milan (Italy) and all the participants signed a written informed consent before enrollment.

## Author Contributions

AS and GG: *in vitro* and *ex vivo* functional and biological characterization of the scDb. FC: production and purification of the scDb and technical support in *in vitro* experiments. FR: choice and supply of ascitic fluids and tumor samples. AS, GG, MF, AA, BF, NZ, and DM: data acquisition and analysis, interpretation of data, and drafting of the manuscript. MF, AS, AG, FR, AA, and MD: conception and design of the study.

### Conflict of Interest

The authors declare that the research was conducted in the absence of any commercial or financial relationships that could be construed as a potential conflict of interest.

## Abbreviations

BiTE: bispecific T-cell engager
bsAb: bispecific antibody
CAR: chimeric antigen receptor
CFSE: carboxyfluorescein succinimidyl ester
DR5: death receptor 5
E:T: effector to target
FBS: fetal bovine serum
HS: human serum
IFN-γ: interferon-gamma
mAb: monoclonal antibody
MHC: major hystocompatibility complex
PBLs: peripheral blood leukocytes
PBS: phosphate-buffered saline
PHA: phytohaemagglutinin
scDb: single chain diabody
SEC: size exclusion chromatography
TAA: tumor-associated antigen
TNBC: triple-negative breast cancer
sTRAIL: soluble TNF-related apoptosis-inducing ligand
TRAIL-R: TNF-related apoptosis-inducing ligand receptor.
