# Supplementary figures and images for: A Bispecific Antibody to Link a TRAIL-Based Antitumor Approach to Immunotherapy
Source: Front Immunol. 2019 Oct 25;10:2514. doi: 10.3389/fimmu.2019.02514 (PMC6823250; doi:10.3389/fimmu.2019.02514)

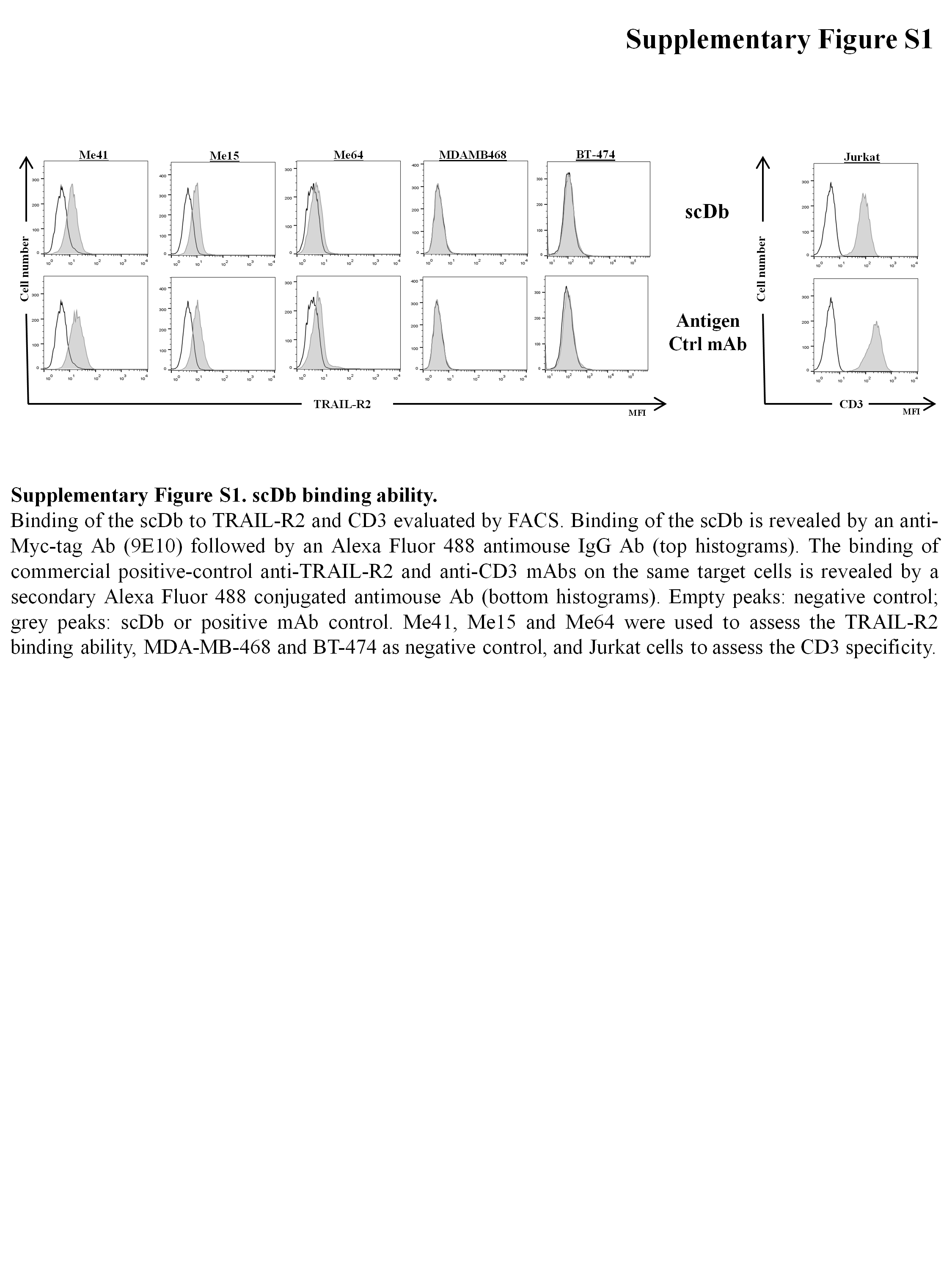

Supplement: Supplementary file 1 [file Image_1.TIF]

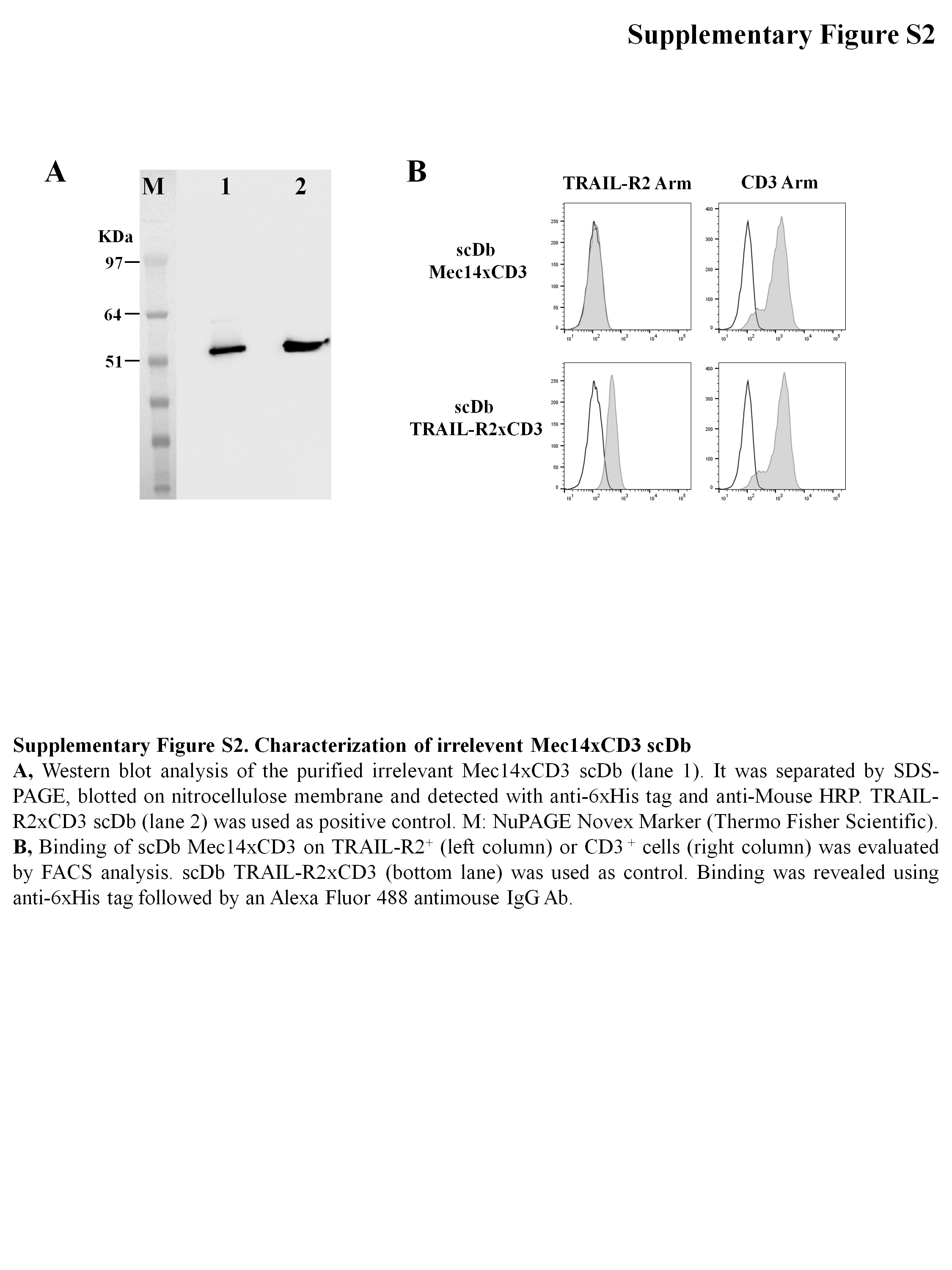

Supplement: Supplementary file 2 [file Image_2.TIF]

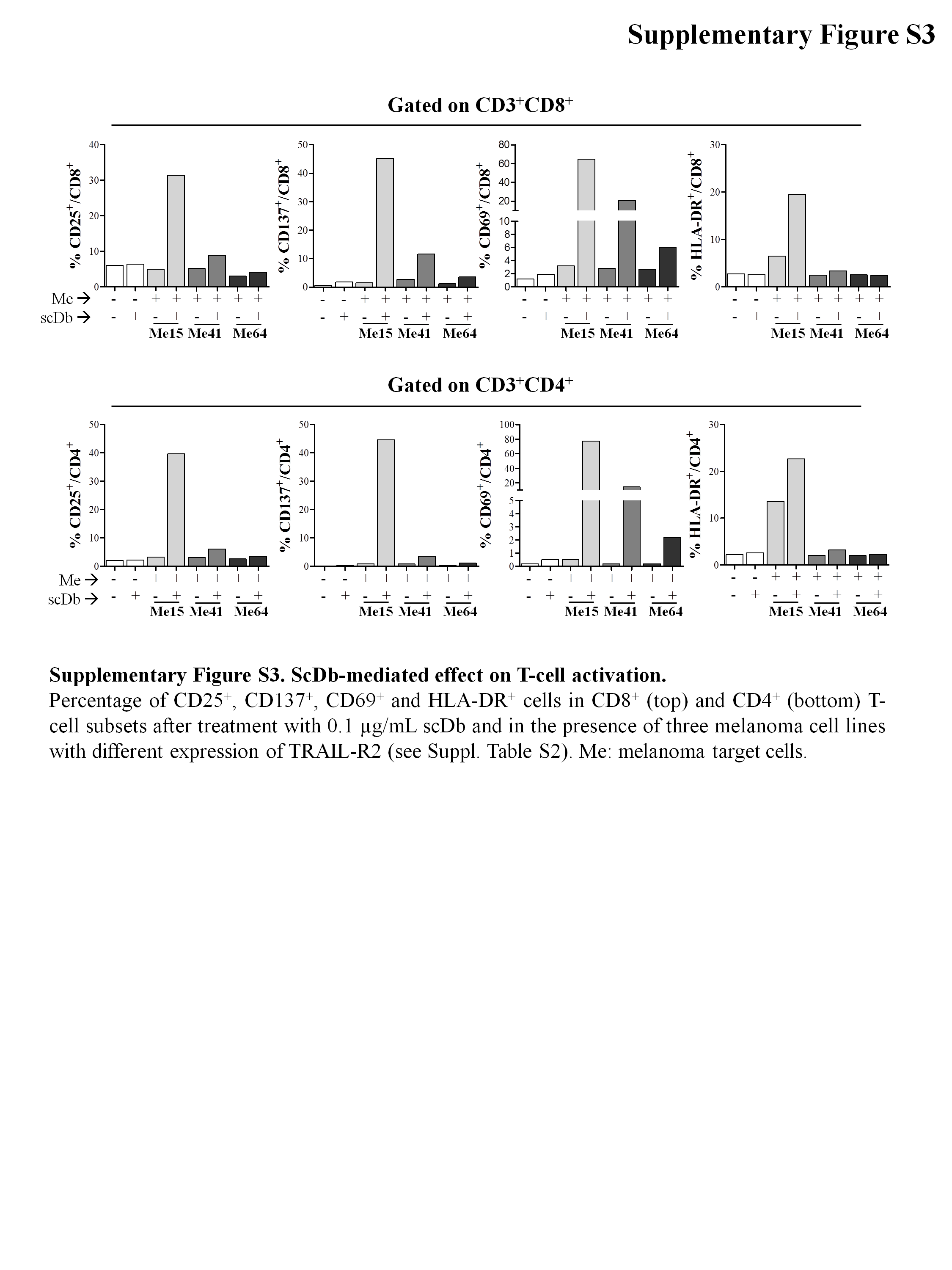

Supplement: Supplementary file 3 [file Image_3.TIF]

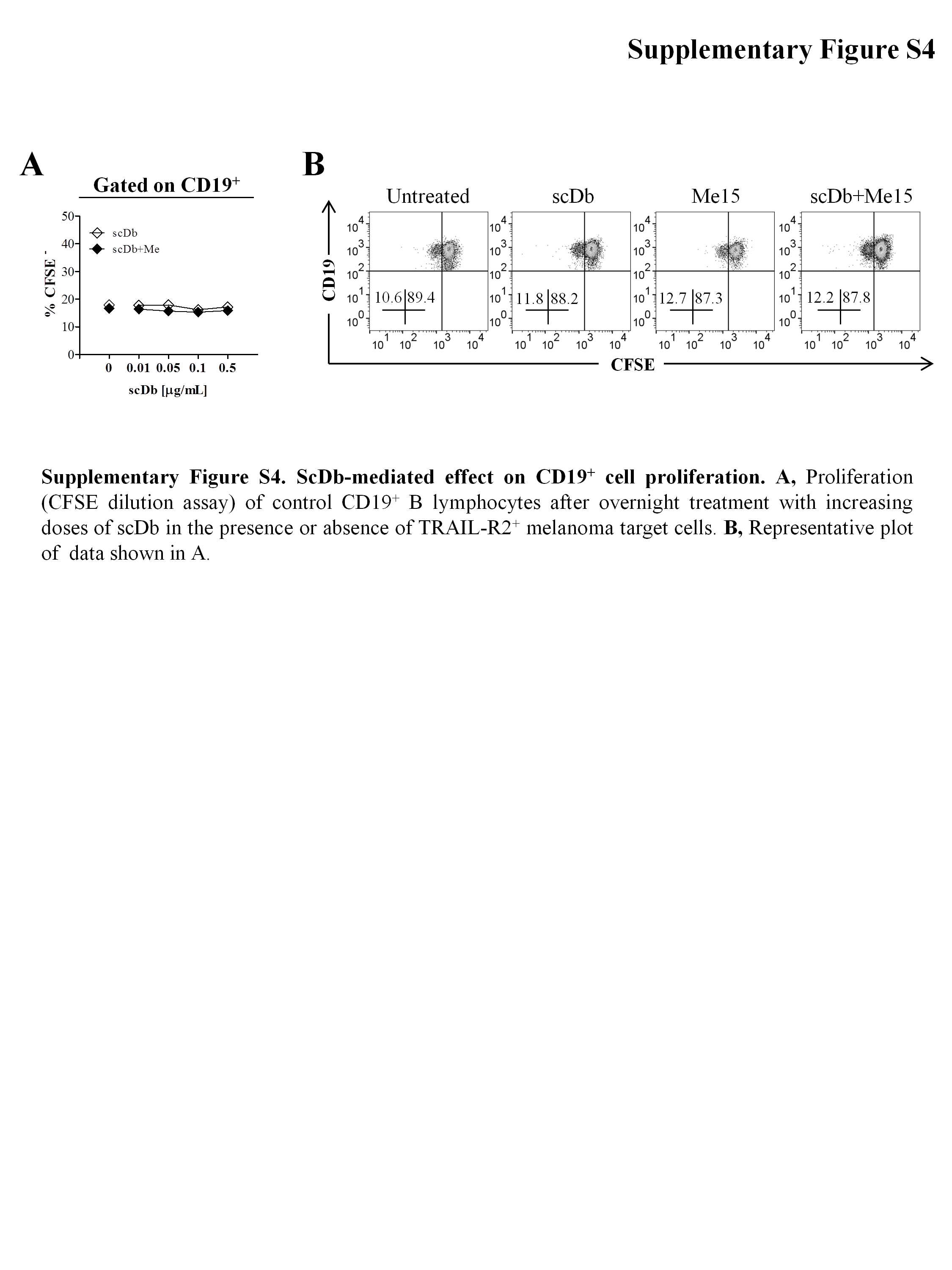

Supplement: Supplementary file 4 [file Image_4.TIF]

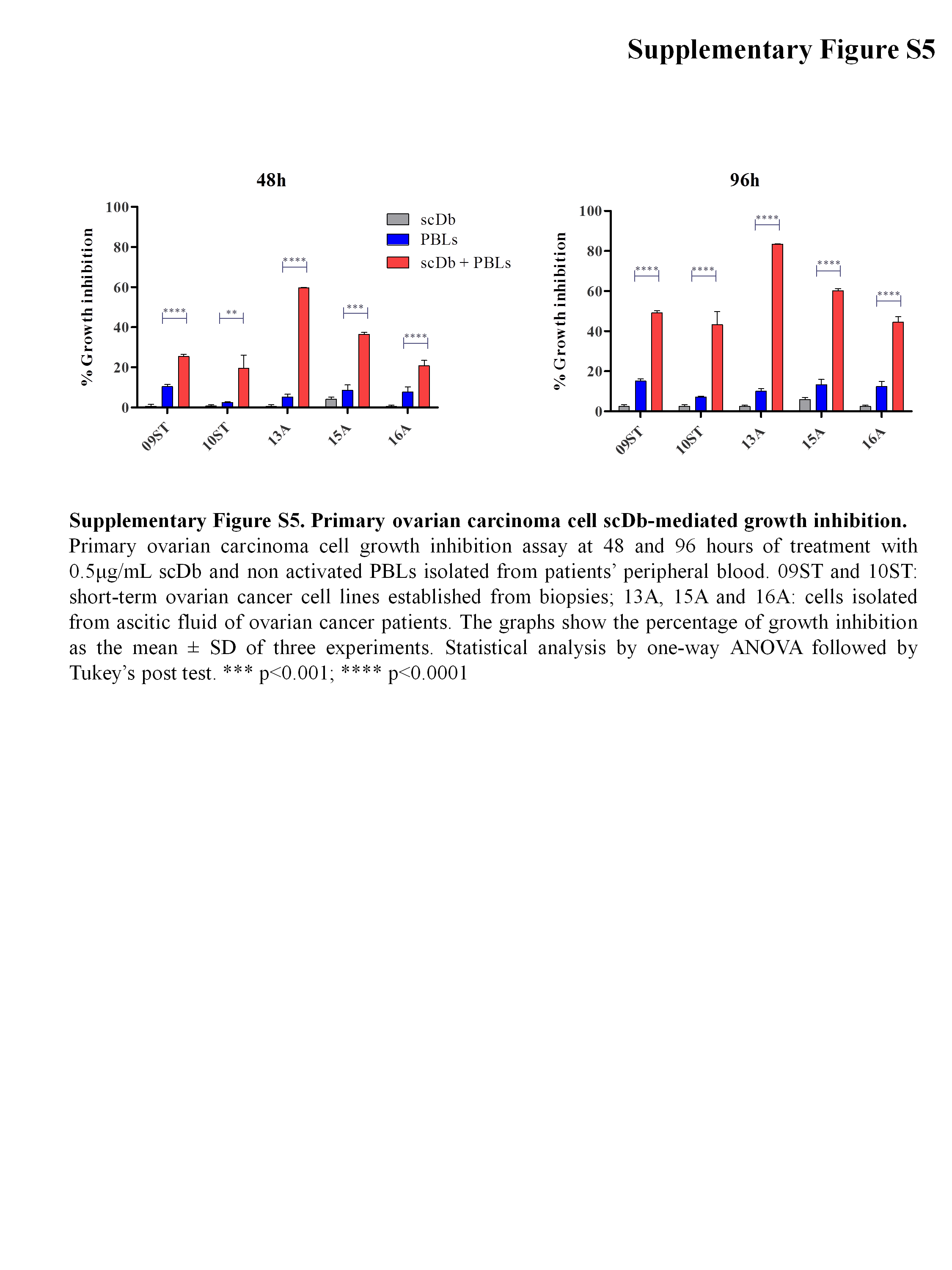

Supplement: Supplementary file 5 [file Image_5.TIF]

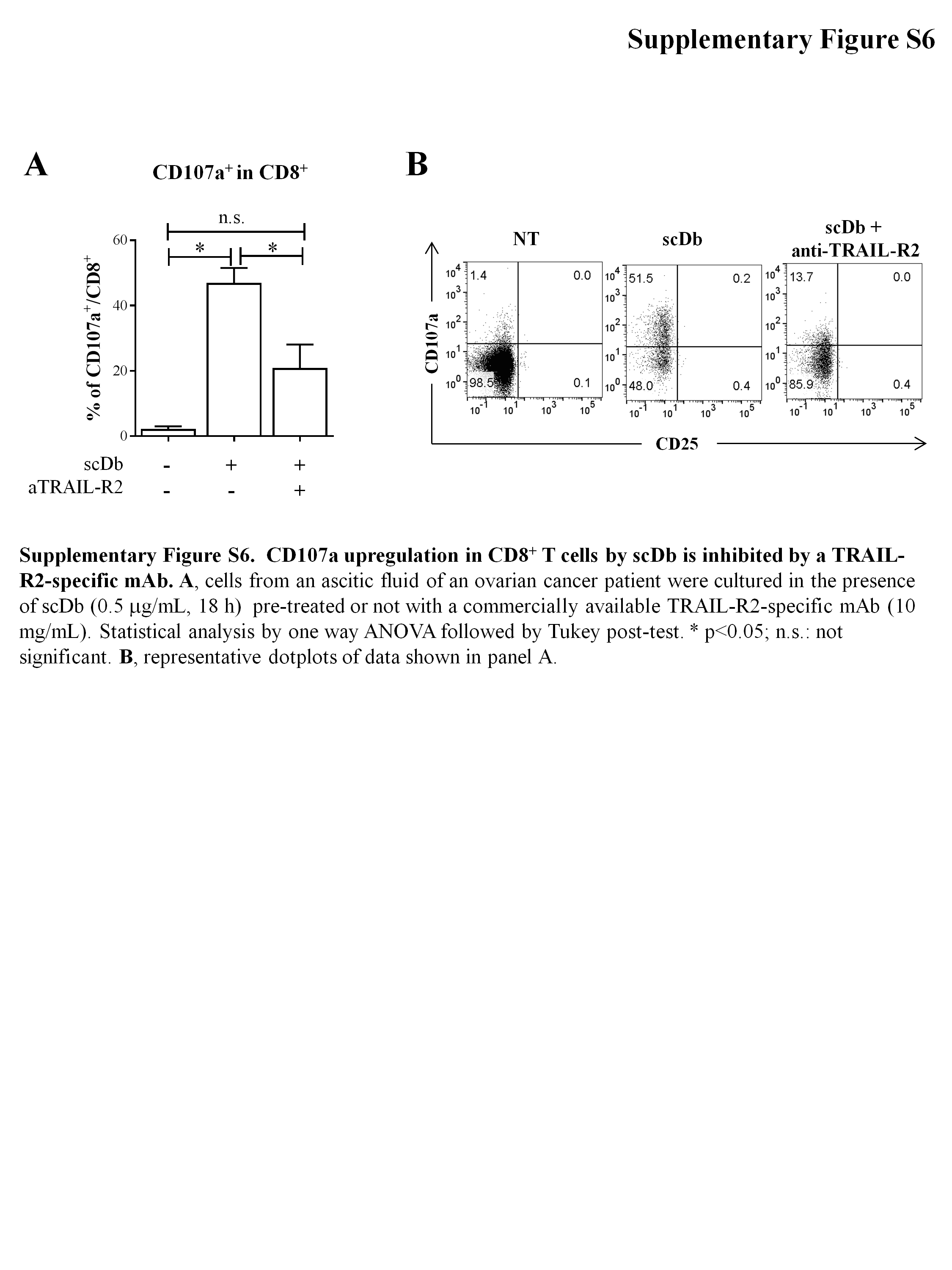

Supplement: Supplementary file 6 [file Image_6.TIF]

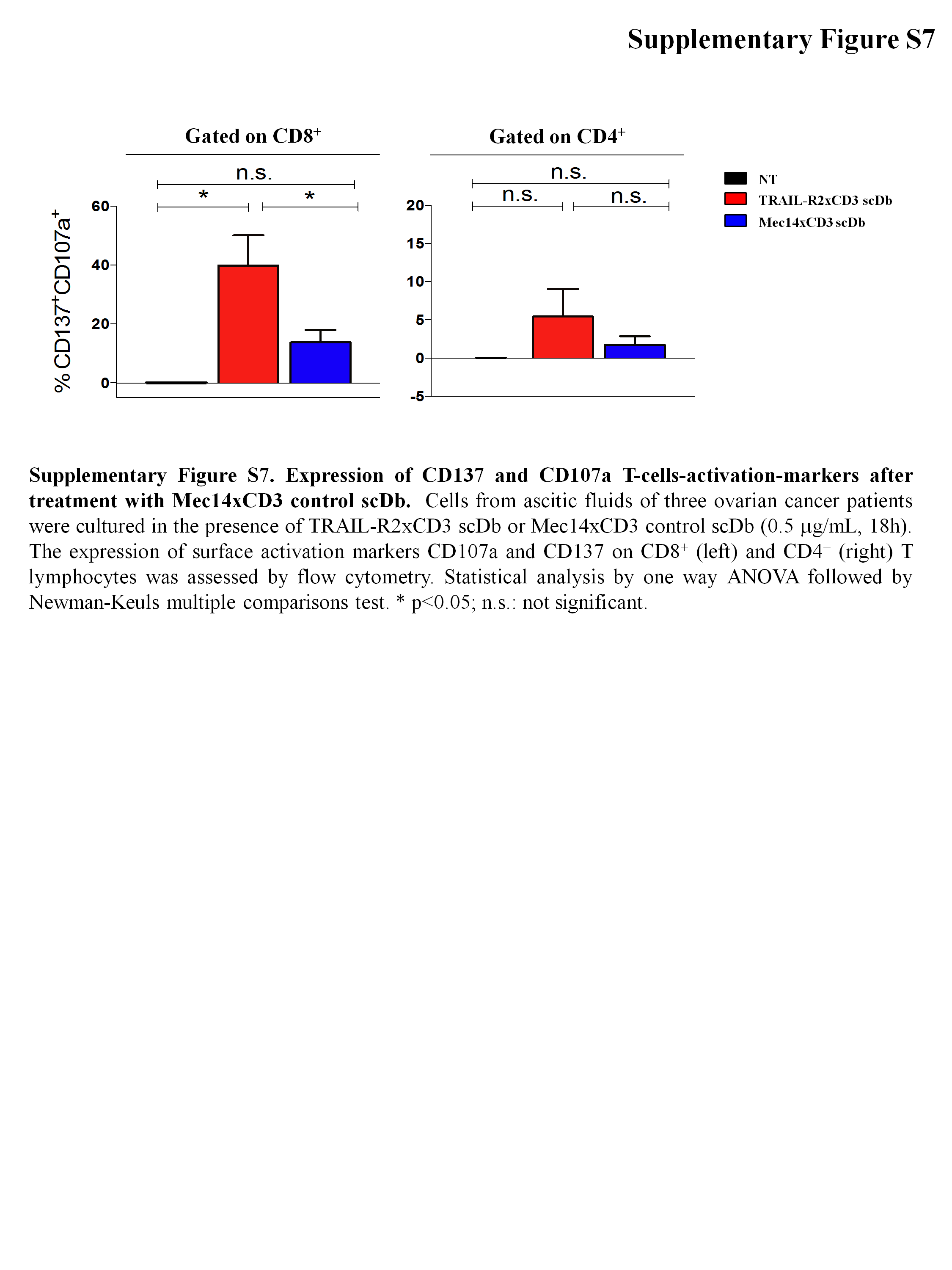

Supplement: Supplementary file 7 [file Image_7.TIF]

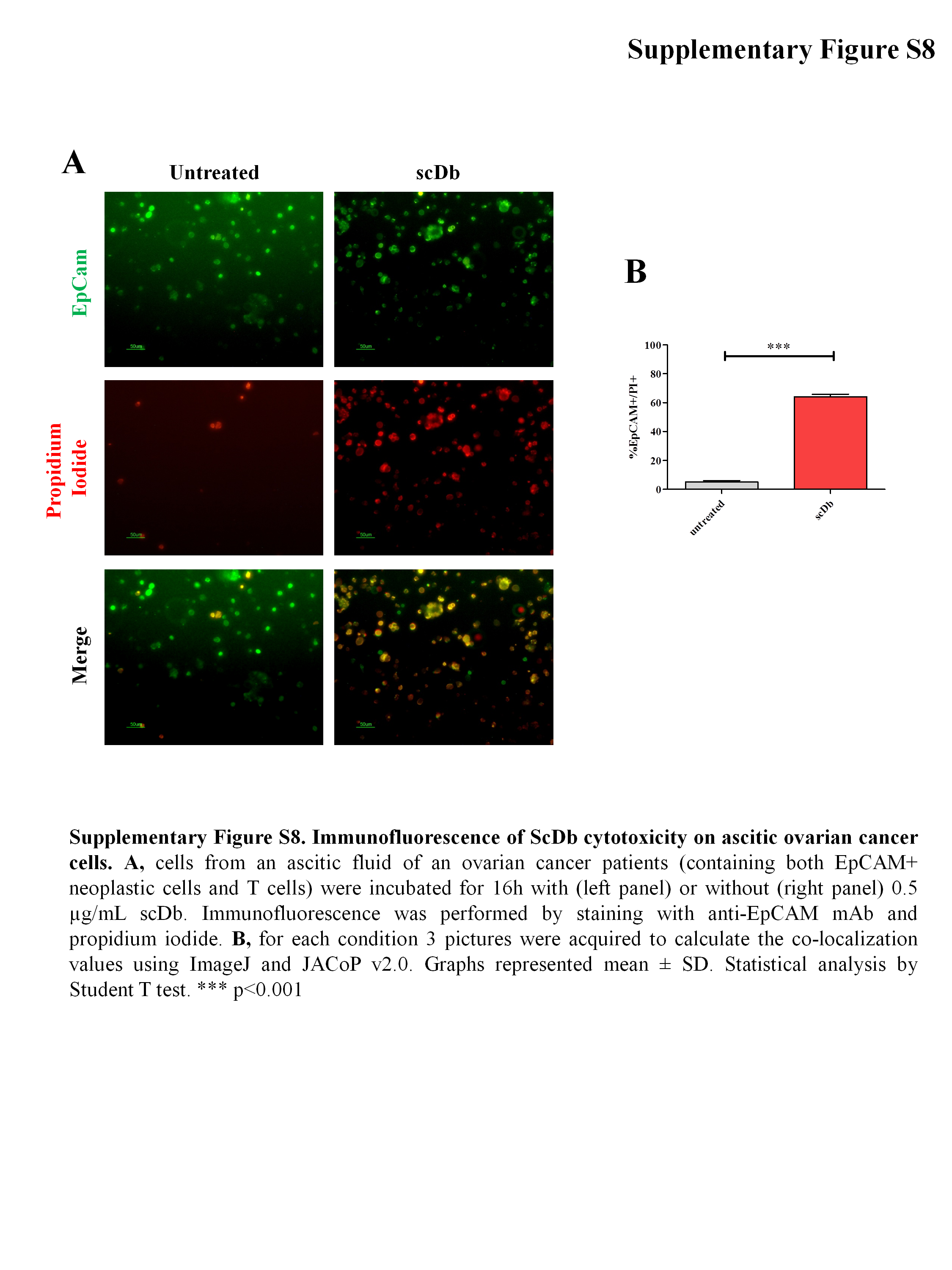

Supplement: Supplementary file 8 [file Image_8.TIF]
